# Sexual and reproductive health outcomes among female sex workers in Johannesburg and Pretoria, South Africa: Recommendations for public health programmes

**DOI:** 10.1186/s12889-017-4346-0

**Published:** 2017-07-04

**Authors:** Mariette Slabbert, Francois Venter, Cynthia Gay, Corine Roelofsen, Samanta Lalla-Edward, Helen Rees

**Affiliations:** 1 Wits Reproductive Health and HIV Institute (Wits RHI), Faculty of Health Sciences, University of the Witwatersrand, Johannesburg, South Africa; 2 University of North Carolina, Chapel Hill, NC USA; 3 Foundation for Professional Development, Pretoria, South Africa

**Keywords:** Female sex workers, HIV, South Africa, Prevention, Sexually transmitted infections, Sexual and reproductive health

## Abstract

**Background:**

The sexual and reproductive health (SRH) status of female sex workers is influenced by a wide range of demographic, behavioural and structural factors. These factors vary considerably across and even within settings. Adopting an overly standardised approach to sex worker programmes may compromise its impact on some sub-groups in local areas.

**Methods:**

Records of female sex workers attending clinic-, community-, or hotel-based health services in Johannesburg (*n* = 1422 women) and Pretoria (*n* = 408 women), South Africa were analysed. We describe the population’s characteristics and identified factors associated with sexual and reproductive health outcomes, namely HIV status; previous symptomatic sexually transmitted infection (STI); modern contraceptive use and number of child dependents.

**Results:**

The women in Johannesburg were less likely than those in Pretoria to have HIV (42.2% vs 52.9%), or previous symptomatic STIs (44.3% vs. 8.3%), and were 1.4 fold less likely to have child dependents (20.1% vs. 15.3%). About 43% of women in Johannesburg were Zimbabwean and 40% in Pretoria. Of concern, only about 15% of women in both sites were using modern contraceptives. Johannesburg women were also more likely to access health services at a hotel (85.0% vs. 80.6%) or clinic (5.7% vs. 0.5%), to have completed secondary education (57.1% vs. 36.0%), and moved house more than twice during the past year (19.6 vs. 2.0%). In both cities, risk of HIV rose rapidly with age (23.8%–58.2% vs. 22.0%–64.8%). Of interest, HIV prevalence was considerably higher in those with consistent condom use with one’s main partner than inconsistent users.

**Conclusions:**

Sex worker populations are heterogeneous. Local health programmes must prioritise services that reflect the variety and complexity of sex worker needs and behaviours, and should be designed in consultation with sex workers. Segmenting sex worker populations according to age, country of origin and place of service delivery, and training healthcare providers accordingly, could help prevent new HIV infections, improve adherence to antiretroviral treatment and increase uptake of SRH services.

## Background

Female sex workers face many barriers to accessing sexual and reproductive health (SRH) care because of stigma and discrimination [1, 2], which increase their vulnerability and impede their right to access health services [3, 4]. Other factors contributing to poor SRH outcomes include high sexually transmitted infections (STI) prevalence [1], HPV infection and thus risk for cervical cancer [5], unintended pregnancies [6, 7], repeated physical and emotional abuse [8], high mobility and frequently an illegal immigrant status [2, 9]. In most countries the prevalence of HIV is 10 to 20 times higher among female sex workers than it is among women in the general population [10–12]. In South Africa, at least one-third of sex workers are HIV positive by the age of 24 and levels of infection reach 80% among older age women [1]. Further, HIV transmission between female sex workers and their clients accounts for an estimated 6–20% of all heterosexual transmission in South Africa. Overcoming these barriers through improved service delivery to link sex workers to early antiretroviral treatment is essential if the ambitious global goal of ending the HIV epidemic by 2030 is to be reached [13].

Most sex work programmes in Africa are not linked with broader HIV care and treatment networks [2]. The South African government recently adopted a comprehensive national HIV plan for sex workers [14]. This plan combines the rollout of pre-exposure prophylaxis (PrEP) for those that are HIV negative, and immediate antiretroviral treatment for HIV-positive sex workers, in addition to six integrated support packages of care encompassing the multi-faceted lives of sex workers. The plan targets SRH and HIV, and includes a minimum package of health services, peer-led service delivery, psychosocial and human rights support, building sex worker organisations, and promoting career paths and economic opportunities. SRH services included in the plan are periodic presumptive treatment for STIs, contraception (including dual protection and emergency contraception), referral for termination of pregnancy, and annual PAP smears for screening for cervical cancer [14].

In a resource-limited setting like South Africa it is important to implement evidence-based care at sufficient scale and tailored to the needs of sex workers in different settings [15]. The objective of this study was to describe the socio-demographic and behavioural characteristics of sex workers in two cities, and to identify risk factors for adverse SRH outcomes. These findings may guide the formulation of more focused and locally-relevant health and social responses in the rollout of the South African HIV plan for sex workers.

## Methods

### Study setting and programme description

In 1995, the Wits Reproductive Health and HIV Institute (Wits RHI) established services for female sex workers in Hillbrow, Johannesburg, South Africa, an area with a long-standing sex worker industry and a popular destination for newly-arrived migrants [16]. Then, in 2014, the Wits RHI set up sex worker services and outreach programmes in Pretoria, the capital city of South Africa, about 50 km from Hillbrow. In both places, peer educators recruit sex workers who can then access a comprehensive package of health education, HIV and SRH services. These services are provided through stand-alone sex worker clinics, mobile vans that deliver clinical services to street-based sex workers within the community, and clinical teams that provide health services in brothels and hotels where sex workers operate.

### Study design

We conducted a retrospective analysis of routinely collected data from the female sex worker programs from 2014 to 2015 in Johannesburg and Pretoria. In both sites, data were collected using standardised forms that were completed when sex workers accessed services for the first time at the clinic, mobile van, hotels or brothels. Data were collected on demographics and sex work history, as well as sexual behaviours with different types of partners and substance use. Clinical staff documented the findings of the physical examination and the woman’s HIV status. Data were included for all female sex workers who attended the services during the study periods.

### Study measures

#### Outcome measures

Four self-reported measures were selected as indicators of sex workers’ SRH status and needs for services: HIV status, previous symptomatic STI other than HIV, modern contraceptive use (using a contraceptive method other than condoms), and not having child dependents (sex workers who reported that no children depend on her income).

#### Independent variables

Demographic variables included age, country of birth, education level, number of adult dependents, number of children living with the sex worker and whether they have a main partner. ‘Main partner’ is defined here as a regular sexual partner with whom the sex worker has a stable relationship and who does not pay for sex.

Behavioural variables included, number of sexual encounters in the last seven days, number of years in sex work, whether they used condoms consistently with their main partners and clients, alcohol and marijuana use, number of times the woman moved house in the last 12 months, and the site of service delivery. ‘Site of delivery’ indicates where the sex worker accessed healthcare for the first time, which could be a clinic, mobile van, or from a hotel or brothel (the latter two grouped together).

### Analysis

Data were extracted from participant files into a Microsoft Access database and exported to STATA 13.1 for analysis. Chi-square tests were used to test relationships between categorical variables and Student *t*-tests were performed on continuous variables. We used multivariate logistic regression to assess associations between the independent and outcome variables. Results are consistently reported for Johannesburg first and then Pretoria.

## Results

The Johannesburg database recorded 1422 first visits and the Pretoria site 408 (Table 1).

**Table 1:** Comparison between demographic and behavioural characteristics of sex workers in Johannesburg and Pretoria

	JOHANNESBURG	PRETORIA	ODDS RATIO	*P* value
	*N* = 1422 N (%)	*N* = 408 N (%)		
HIV positive total population	575/1364 (42.2)	211/399 (52.9)	0.6 (0.5–0.8)	<0.001
^*^South Africans	210/593 (35.4)	80/158 (50.6)		
^*^Zimbabweans	286/589 (48.5)	123/229 (58.3)		
^*^Other origin	79/181(43.6)	8/12 (66.7)		
Previous STI**	630/1422 (44.3)	34/408 (8.3)	8.8 (6.0–13.0)	<0.001
Use modern contraception	187/1422 (15.1)	62/408 (15.2)	0.9 (0.6–1.2)	0.288
No child dependents	281/1401 (20.1)	61/399 (15.3)	1.4 (1.0–1.9)	0.032
Age (years)				
<25	340/1422 (23.9)	43/404 (10.6)	2.6 (1.9–3.8)	<0.001
25–29	517/1422 (36.4)	92/404 (22.8)	1.9 (1.5–2.5)	
30–34	369/1422 (26.0)	128/404 (31.7)	0.8 (0.6–1.0)	
35+	196/1422 (13.8)	141/404 (34.9)	0.3 (0.2–0.4)	
Education				
Primary	46/1414 (3.3)	61/406 (15.0)	0.2 (0.1–0.3)	<0.001
Grade 8–10	490/1414 (34.7)	180/406 (44.3)	0.7 (0.5–0.8)	
Grade 11–12	808/1414 (57.1)	146/406 (36.0)	2.4 (1.9–3.0)	
Tertiary level	70/1414 (5.0)	19/406 (4.7)	1.1 (0.6–1.9)	
Have a main partner	693/1422 (48.7)	221/406 (54.4)	0.8 (0.6–1.0)	0.043
>1 child lives with sex worker	163/1233 (13.2)	101/408 (24.8)	0.5 (0.3–0.6)	<0.001
≥3 dependent adults	456/1398 (32.6)	153/400 (38.3)	0.8 (0.6–1.0)	0.036
Country of birth				
South Africa	621/1422 (43.7)	234/408 (59.1)	0.6 (0.5–0.7)	<0.001
Zimbabwe	610/1422 (42.9)	162/408 (39.7)	1.1 (0.9–1.4)	
Other	191/1422 (13.4)	12/408 (2.9)	5.1 (2.9–10.2)	
Alcohol use	977/1314 (74.4)	221/390 (56.7)	2.2 (1.7–2.8)	<0.001
Marijuana use	92/1314 (7.0)	32/390 (8.2)	0.8 (0.5–1.3)	0.422
Years in sex work				
<2 2–5 >5	496/939 (52.8) 311/939 (33.1) 132/939 (14.1)	298/407 (73.2) 71/407 (17.4) 38/407 (9.3)	0.4 (0.3–0.5) 2.3 (1.7–3.2) 1.6 (1.1–2.4)	<0.001
Sex encounters past 7 days				
0–10	515/1311 (39.3)	41/218 (18.8)	2.8 (2.0–4.1)	<0.001
11–20	452/1311 (34.5)	72/218 (33.0)	1.1 (0.8–1.5)	
>20	344/1311 (26.2)	105/218 (48.2)	0.4 (0.3–0.5)	
Consistent condom use: main partner	201/1422 (14.1)	58/208 (27.9)	0.4 (0.3–0.6)	<0.001
Consistent condom use: clients	1289/1302 (99.0)	359/382 (94.0)	6.4 (3.0–13.8)	<0.001
Times moved house past year				
0	729/1422 (51.3)	336/408 (82.4)	0.2 (0.2–0.3)	<0.001
1	415/1422 (29.2)	64/408 (15.7)	2.2 (1.6–3.0)	
2	278/1422 (19.6)	8/408 (2.0)	24.3 (9.3–90.3)	
Site receives services				
Hotel	1129/1328 (85.0)	329/408 (80.6)	1.4 (1.0–1.8)	<0.001
Clinic	75/1328 (5.7)	2/408 (0.5)	12.2 (3.2–102.5)	
Mobile vans	124/1328 (9.3)	77/408 (18.9)	0.4 (0.3–0.6)	

Numbers in brackets give the proportion of the total population. N could differ across rows because of missing data.

Adjusted Odds Ratio (AOR) and accompanying *P* value is provided for each sub group, compared to the rest of the group, thus, for example, the proportion working in a hotel is compared to women working in all other settings

^*^Breakdown of positivity rate by country

STI Sexually Transmitted Infection

### Socio-demographics and sexual behaviours

Compared with sex workers in Pretoria, those in Johannesburg were almost 5 years younger (Johannesburg mean = 28.6 sd = 5.35, Pretoria mean = 33.2 sd = 9.9, *P* < 0.001). A third (34.9%) of the sex workers in Pretoria were above 35 years of age compared to only 13.8% in Johannesburg (*P* < 0.001) (Table 1). Women in Johannesburg were more likely than those in Pretoria to have finished secondary education (57.1% vs. 36.0%, OR = 2.4 *P* = 0.021) and were half as likely to have more than one child living with them (13.2% vs. 24.8%, OR = 0.5; *P* < 0.001). In both sites, around half of the women had a main partner (48.7% vs. 54.4%, *P* = 0.043) and a third (32.6% vs. 38.3%, *P* = 0.036) had more than 3 adult dependents. Almost half of the sex workers in Johannesburg were from Zimbabwe (43.7%) and 13.4% from countries other than South Africa and Zimbabwe, while only 39.7% were from Zimbabwe in Pretoria (*P* = 0.005) and 2.9% from other countries.

Alcohol use was twice as high among women in Johannesburg as in Pretoria (74.4% vs. 56.7%, OR = 2.2; *P* < 0.001), but in both sites around 8% of women reported marijuana use. Johannesburg women moved house markedly more often in the past year (mean 3.7 vs 1.3; *P* < 0.001), with women in Johannesburg 24 times more likely than those in Pretoria to have moved house more than twice in the last year (CI = 9.3–90.3). Women in Johannesburg were almost 3 times more likely to have had less than 10 sexual encounters in the past 7 days (OR = 2.8, CI 2.0–4.1), while women in Pretoria were twice as likely as those in Johannesburg to have had more than 20 encounters (OR = 0.4, CI = 0.3–0.5, *P* < 0.001). Even though they were younger on average, sex workers from Johannesburg had engaged in sex work for more years than their counterparts in Pretoria, with those in Johannesburg twice as likely than those in Pretoria to have been in sex work for longer than two years (OR = 2.3, CI = 1.7–3.2). Women in Johannesburg reported higher condom use with commercial clients (99.0% vs. 94.0%, OR = 6.4, *P* < 0.001), but lower condom use with main partners (14.1 vs. 27.9%, OR = 0.4, *P* < 0.001). In both sites, consistent condom use with main partners was three to seven-fold lower than condom use with commercial clients.

### Factors associated with HIV status and symptomatic STIs

For SRH outcomes at the first visit, a lower proportion of women in Johannesburg than in Pretoria (42.2% and 52.9%) tested HIV positive (OR = 0.6; *P* < 0.001) and they reported more previous symptomatic STI (44.3% and 8.3%, OR = 8.8; *P* < 0.001) (Table 1).

In both sites, odds of HIV infection rose stepwise with each increase in age category. Women over 35 were 2-times more likely to be HIV positive than those under 25 (Table 2). In Johannesburg, Zimbabwean nationality (AOR = 0.6, CI = 0.5–0.8) and having Grade 11 or higher education level were associated with being HIV negative.

**Table 2:** Association between demographics and behavioural factors with HIV status and STIs among sex workers in Johannesburg and Pretoria

	HIV POSITIVE				PREVIOUS STI			
*N* = 1422 JHB *N* = 408 PTA	JHB N (%)	Multivariate analysis AOR (95% CI)	PTA N (%)	Multivariate analysis AOR (95% CI)	JHB N (%)	Multivariate analysis AOR (95% CI)	PTA N (%)	Multivariate analysis OR (95% CI)
SOCIO DEMOGRAPHICS								
Age at 1st visit (years)								
<25 25–29 30–34 >35	77 (23.8)*** 206 (41.6) 182 (51.1) 110 (58.2)	1.0 1.0 (0.8–1.2) 1.6 (1.3–2.1) 2.1 (1.5–2.9)	9 (22.0)*** 36 (40.5) 73 (57.9) 90 (64.8)	1.0 0.5 (0.3–0.9) 1.4 (0.9–2.1) 2.1 (1.4–3.4)	167 (49.1) 228 (44.1) 159 (43.1) 76 (38.8)		5 (11.6) 4 (4.4) 14 (10.9) 8 (5.7)	1.0 0.30 (0.1–1.3) 0.58 (0.2–1.9) 0.32 (0.1–1.1)
Education								
Primary Grade 8–10 Grade 11–12 Tertiary level	27 (61.4)** 214 (45.6) 308 (39.6) 21 (31.8)	1.0 1.3 (1.0–1.6) 0.8 (0.6–1.0) 0.6 (0.5–1.1)	37 (62.7)* 98 (56.0) 68 (46.9) 7 (36.8)	1.0 1.3 (0.8–1.9) 0.7 (0.5–1.1) 0.5 (0.2–1.4)	16 (34.8)** 198 (40.4) 384 (47.5) 27 (38.6)	1.0 0.8 (0.6–1.0) 1.4 (1.1–1.7) 0.8 (0.5–1.3)	4 (6.7)** 24 (13.3) 5 (3.4) 1 (5.3)	1.0 1.3 (0.4–5.3) 0.4 (0.1–1.6) 0.2 (0.0–3.2)
Main partner								
No Yes	298 (42.9) 277 (41.5)		103 (57.22) 107 (49.3)		340 (46.6)* 290 (41.9)	1.0 0.8 (0.7–1.0)	12 (6.5) 22 (10.0)	
Child lives with sex worker								
0 ≥1	160 (53.2) 51 (52.0)		160 (53.2) 51 (52.0)				27 (8.8) 6 (5.9)	
Adult dependents								
0 1–2 ≥3	71 (42.3) 321 (43.6) 173 (39.7)		18 (46.2) 112 (55.5) 77 (51.0)		91 (50.8)* 319 (41.8) 211 (46.3)	1.0 0.8 (0.6–1.0) 1.1 (0.9–1.4)	5 (12.8) 14 (6.7) 15 (9.8)	
Country of birth								
South Africa Zimbabwe Other	286 (48.6) *** 210 (35.4) 79 (43.7)	1.0 0.6 (0.5–0.8) 1.1 (0.8–1.5)	123 (53.7) 80 (50.6) 8 (66.7)		252 (41.3) 288 (46.4) 90 (47.1)		12 (5.1)*** 18 (11.1) 4 (33.3)	1.0 1.4 (0.53–3.9) 7.8 (1.6–37.1)
BEHAVIOURS								
Alcohol use								
No Yes	125 (38.9) 406 (43.1)		84 (50.6) 117 (53.7)		157 (46.6) 428 (43.8)		14 (8.3) 18 (8.1)	
Marijuana use								
No Yes	498 (42.4) 33 (37.1)		181 (51.1) 20 (66.7)		542 (44.4) 43 (46.7)		27 (7.5) 5 (15.6)	
Years in sex work								
<2 2–5 >5	180 (38.0)*** 125 (41.8) 76 (60.8)	1.0 1.0 (0.7–1.3) 2.4 (1.6–3.6)	158 (53.9) 33 (48.5) 20 (52.6)		243 (49.0) 134 (43.1) 56 (42.4)		20 (6.7)** 11 (15.5) 2 (5.3)	1.0 2.0 (0.7–5.4) 0.7 (0.1–3.7)
Sex encounters last 7 days								
0–10 11–20 >20	220 (45.1)** 188 (42.9) 122 (36.3)	1.0 1.1 (0.8–1.3) 0.7 (0.6–0.9)	21 (53.9) 38 (53.3) 57 (54.8)		223 (43.3)* 220 (48.7) 140 (40.7)	1.0 1.1 (0.9–1.4) 0.8 (0.6–1.0)	2 (4.9) 6 (8.3) 12 (11.4)	
Main partner condom use								
Every time Never or sometimes No main partner	91 (47.6)** 122 (35.7) 362 (43.6)	1.0 1.3 (1.0–1.8) 0.7 (0.6–1.0)	37 (66.1)** 65 (43.3) 109 (56.5)	1.0 0.5 (0.25–1.02) 0.8 (0.43–1.66)	92 (45.8) 152 (43.3) 386 (44.4)		14 (9.3) 5 (8.6) 15 (7.5)	
Client condom use								
Every time Never or sometimes	522 (42.0) 6 (54.6)		188 (53.1) 9 (40.9)		578 (44.8)** 1 (7.7)	1.0 0.1 (0.0–0.7)	29 (8.1) 3 (13.0)	
Times moved in last year								
0 1 >1	284 (40.7) 180 (45.6) 111 (41.1)		167 (50.8) 38 (61.3) 6 (75.0)		328 (45.0)** 186 (44.8) 116 (41.7)	1.0 1.0 (0.8–1.3) 0.9 (0.7–1.2)	24 (7.1)* 8 (12.5) 2 (25.0)	1.0 1.67 (0.66–4.23) 5.0 (0.77–33.12)
Site received services								
Hotel Clinic Mobile van	425 (39.2)** 36 (49.3) 66 (55.0)	1.0 1.4 (0.9–2.3) 1.8 (1.2–2.7)	172 (53.3) 1 (100.0) 38 (50.7)		511 (45.3) 27 (36.0) 55 (44.3)		27 (8.2) 0 (0.0) 7 (9.1)	

***P < 0.001 **P < 0.05 *P = 0.05–0.1

Statistical tests compare distribution of outcomes in each site, does not compare sites.

AOR Adjusted odds ratio

STI Sexually Transmitted Infection

In Johannesburg, more than 5 years in sex work (AOR = 2.4, CI = 1.6–3.6)) and accessing services from a mobile unit, as opposed to a hotel (AOR = 1.8, CI = 1.2–2.7) showed a positive association with having HIV. Of interest, HIV prevalence was considerably higher in those with consistent condom use with one’s main partner than inconsistent users in univariate analysis. The association was, however, only detected in the multivariate model of the Johannesburg site.

In Pretoria, sex workers from Zimbabwe and other countries were more likely to have reported a prior STI. Also in that city, inconsistent condom use with clients was associated with a lower odds of an STI. In Johannesburg, those with Grade 11–12 education had higher STI levels than women with only primary schooling.

### Contraception coverage and number of child dependents

Women in both sites had low levels of use of modern contraceptives (around 15%) (Table 1). In both sites, the majority of women reported using condoms as their preferred method of contraception (80.6% and 72.8%). In Johannesburg, modern contraceptive use was highest among those with no adult dependents (17.9% vs. 10.3% in those with more than one) and highest among women from Zimbabwe and other countries (12.4%, 21.5%) than South African women (11.3%) (Table 3). Of the 65 (1.9%) women that reported being pregnant at the time of their visit, almost 40% did not plan to continue with the pregnancy (not shown).

**Table 3:** Association between demographics and behavioural factors with contraceptive use other than condoms and having child dependents among sex workers in Johannesburg and Pretoria

	USE MODERN CONTRACEPTION				NO CHILD DEPENDENTS			
*N* = 1422 *N* = 408 PTA	JHB N (%)		PTA N (%)	Multivariate analysis AOR (95% CI)	JHB N (%)	Multivariate analysis OR (95% CI)	PTA N (%)	Multivariate analysis AOR (95% CI)
SOCIO DEMOGRAPHICS								
Age at 1st visit								
<25 25–29 30–34 >35	40 (11.8) 73 (14.1) 50 (13.6) 24 (12.2)		7 (16.3) 14 (15.2) 18 (14.1) 22 (15.6)		104 (31.4)*** 98 (19.1) 54 (14.9) 25 (12.9)	1.0 0.9 (0.7–1.2) 0.6 (0.4–0.9) 0.5 (0.3–0.9)	14 (35.0)** 11 (12.1) 16 (12.8) 20 (14.3)	1.0 0.7 (0.3–1.4) 0.7 (0.4–1.4) 0.9 (0.5–1.6)
Education								
Primary Grade 8–10 Grade 11–12 Tertiary level	5 (10.9) 71 (14.5) 97 (12.0) 11 (15.7)		7 (11.5) 25 (13.9) 25 (17.1) 3 (15.8)		11 (23.9)** 107 (22.2) 140 (17.6) 20 (28.6)	1.0 1.2 (0.9–1.6) 0.7 (0.5–0.9) 1.7 (0.9–2.9)	10 (16.7) 23 (13.0) 25 (17.5) 3 (15.8)	
Main partner								
No Yes	106 (14.5) 81 (11.7)		29 (15.7) 33 (14.9)		149 (20.6) 132 (19.4)		23 (12.9) 38 (17.4)	
>1 child lives with sex worker								
0 ≥1	239 (19.7) 42 (22.6)		45 (14.7) 17 (16.8)		117 (11.0) 23 (14.1)		61 (20.5)*** 0 (0.0)	1.0 0 (0–0.1)
Adult dependents								
0 1–2 ≥3	32 (17.9)** 85 (11.1) 68 (14.9)	1.0 0.7 (0.5–0.9) 1.2 (0.9–1.7)	4 (10.3) 30 (14.4) 22 (14.4)		63 (35.2)*** 132 (17.3) 86 (18.9)	1.0 0.7 (0.5–0.9) 0.9 (0.7–1.2)	11 (28.2)** 31 (15.0) 19 (12.5)	1.0 0.9 (0.5–1.7) 0.7 (0.4–1.3)
Country of birth								
South Africa Zimbabwe Other	69 (11.3)** 77 (12.4) 41 (21.5)	1.0 1.5 (1.0–2.3) 1.2 (0.9–1.7)	36 (15.4) 25 (15.4) 1 (8.3)		130 (21.8)** 106 (17.2) 45 (23.9)	1.0 0.7 (0.5–1.0) 1.3 (0.9–1.9)	41 (17.9)** 16 (10.1) 4 (36.4)	1.0 0.5 (0.3–0.9) 2.5 (0.4–11.3)
BEHAVIOURS								
Alcohol use								
No Yes	44 (13.1) 122 (12.5)		25(14.8) 32 (14.5)		56 (16.8) 196 (20.4)		20 (12.1) 39 (18.0)	
Marijuana use								
No Yes	151 (12.4) 15 (16.3)		54 (15.1) 3 (9.4)		228 (18.9)** 24 (27.0)	1.0 1.6 (0.9–2.6)	49 (14.0)** 10 (31.3)	1.0 2.8 (1.1–6.6)
Years in sex work								
<2 2–5 >5	72 (14.5) 37 (11.9) 20 (15.2)		50 (16.8) 8 (11.3) 4 (10.5)		100 (20.3) 59 (19.2) 29 (22.1)		45 (15.6) 11 (15.5) 5 (13.2)	
Sex encounters last 7 days								
0–10 11–20 >20	62 (12.0) 56 (12.4) 48 (14.0)		4 (9.8) 16 (22.2) 18 (17.1)		102 (20.1) 84 (18.9) 65 (19.1)		9 (22.0) 7 (10.3) 14 (13.9)	
Main partner condom use								
Every time Never or sometimes No main partner	26 (12.9) 44 (12.5) 117 (13.5)		11 (19.0) 18 (12.0) 33 (16.5)	1.0 0.5 (0.3–1.0) 0.8 (0.4–1.7)	34 (17.2) 70 (20.5) 177 (20.6)		12 (21.1) 26 (17.6) 23 (11.9)	
Client condom use								
Every time Never or sometimes	160 (12.4)** 5 (38.5)	1.0 4.4 (1.1–15.5)	50 (13.9) 4 (17.4)		246 (19.4)* 5 (38.5)	1.0 2.6 (0.7–9.1)	47 (13.4)** 8 (34.8)	1.0 3.5 (1.2–9.2)
Times moved in last year								
0 1 >1	82 (11.3)*** 78 (18.8) 27 (9.7)	1.0 1.9 (1.4–2.6) 0.7 (0.4–1.0)	53 (15.8) 8 (12.5) 1 (12.5)		130 (18.2) 85 (20.6) 66 (24.2)		52 (15.8) 9 (14.5) 0 (0.0)	
Site receives services								
Clinic Hotel Mobile van	14 (18.7) 138 (12.2) 18 (14.5)		48 (14.6) 1 (50.0) 13 (16.9)		15 (20.0)** 240 (21.6) 9 (7.6)	1.0 1.9 (1.2–3.2) 0.3 (0.1–0.6)	0 (0.0) 45 (13.9) 16 (21.6)	

***P < 0.001 **P < 0.05 *P = 0.05–0.1

Statistical tests compare distribution of outcomes in each site, does not compare sites

AOR Adjusted odds ratio

STI Sexually Transmitted Infection

Sex workers in Johannesburg were more likely to have no child dependents than Pretoria women (20.1% vs. 15.3%, OR = 1.4; CI = 1.0–1.9) (Table 1). Predictably, younger age (Johannesburg AOR = 0.5, CI = 0.3–0.9, Pretoria AOR = 0.9, CI = 0.5–1.6) showed positive relationships with not having child dependents in both sites (Table 3). There was no association between contraceptive use and number of child dependents.

Women from Zimbabwe (Johannesburg AOR = 0.7, CI = 0.5–1.0; Pretoria AOR = 0.5, CI = 0.3–0.9) were less likely to have no child dependents than South African women. Women with no child dependents were more likely to smoke marijuana (Johannesburg AOR = 1.6, CI = 0.9–2.6, Pretoria AOR = 2.8, 1.1–6.6). In Johannesburg, compared to women attending the clinic, those with no child dependents were twice as likely to access services from a hotel (AOR = 1.9, CI = 1.2–3-2) and 70% less likely (AOR = 0.3, CI = 0.1–0.6) from a mobile unit.

## Discussion

This study, conducted in two urban sites in Gauteng Province, shows that female sex workers are a very heterogeneous group, and have quite disparate needs [6, 17], both within and between sites. Sex workers might have varied motivations for entering the profession [18]; with many women supporting themselves and their dependants through sex work [19]. Poverty, large numbers of dependents, lack of other employment options might increase the risk taking behaviours of these women [18]. With high HIV prevalence in the sex worker population, PrEP for HIV-negative sex workers and immediate antiretroviral treatment for HIV-positive women must be standard practice, as stipulated within the South African sex worker HIV plan. Screening for STIs is also a key part of sex worker services.

Women in Johannesburg had lower levels of HIV, but self-reported STIs were much more common. They were younger, better educated, and were more likely to come from outside South Africa than those in Pretoria. They were also twice as likely to drink alcohol, had been in sex work for longer than their colleagues in Pretoria, but had fewer sex encounters in the last seven days. On the domestic front, women in Johannesburg were less likely to have a main partner, less likely to have child dependents and more likely to have moved house in the last year. These differences were associated with SRH outcomes and require differentiated sex work-specific preventive health care [1, 16, 20].

As in other studies [1], younger age was linked with lower HIV positivity and less child dependents. To prevent new HIV infections and unintended pregnancy, young sex workers constitute a key group to be targeted with HIV interventions (such as PrEP and condom negotiation skills, especially with main partners) as well as modern contraception services. Levels of self-reported STIs in young women were high, raising their risk for HIV acquisition. This presents an argument for the use of periodic presumptive treatment for STIs and PrEP [1, 21]. In contrast, the emphasis of services for women older than 25, those who have been in sex work for some time and those who are already HIV-positive must be on psychosocial support and motivation to stay adherent to treatment regimens [4]. Given their experience in the trade and to complement age-matched peer educators, older sex workers might be trained to provide mentoring for younger sex workers, assisting them to lower the risks inherent in the industry [22].

The finding that sex workers in Johannesburg are younger, yet have been in sex work longer than their counterparts in Pretoria could be ascribed to several factors and was observed in other settings where groups of sex workers were compared [9]. Firstly, the city centre in Johannesburg is renowned for commercial sex work and women who exchange sex for money may more readily adopt a sex worker identity than those in Pretoria [23]. If sex-for-money exchange in Pretoria were initially understood as a way to survive financially and not as formal ‘sex work’, these women might be less likely to report intermittent periods of ‘sex-for-money to survive’ as time doing sex work and to access services labelled as being for sex workers [24]. Secondly, the majority of women in Johannesburg migrated from Zimbabwe or other countries, often at a young age. These women might enter sex work early as they lack official documentation and experience xenophobic-related stigma that diminishes their employment opportunities in other sectors [16, 25].

Migrancy and mobility are an inherent part of sex work in most settings [8, 16, 26], which limits their contact with health providers [4, 26]. The reasons for women having to relocate, especially those in Johannesburg, and the impact this may have on income and the welfare of their children are important factors to consider in programming. Support groups and sex worker networks can play a valuable role in advising their fellow sex workers about stable accommodation [20, 23]. Reducing levels of mobility will promote retention in programmes, continuity of care within services such as ART and improve access to familiar health providers.

Sex workers are more open to accessing healthcare if services are delivered at their place of work [2, 23]. This is clearly seen from the several fold higher number of visits held through outreach to hotels and street-based venues, than at the clinics. Site of service delivery has important implications. Firstly, sex workers often only report to the clinic when they have disease symptoms or are already suffering from advanced disease (HIV prevalence was highest among women at the clinic, for example). We propose that sex work programmes use outreach peer educators and peer networks to encourage sex workers to seek preventative services or health care early when symptoms arise. Secondly, sex workers served by the mobile vans are usually street-based and might be more susceptible to violence from clients, police arrest, and have less access to condoms and healthcare than their colleagues in hotels and brothels [6]. Also, homeless women might not have a place to store their medication, even if they were to access treatment. The increased vulnerability of street-based sex workers is reflected in their higher HIV prevalence than hotel-based women. Routine enquiry about violence-related trauma and violation of human rights is important in outreach, but also in other service sites [17].

Health programming often only caters for occupational hazards, like unprotected sex with commercial clients [8] and substance use [27], omitting the compounding exposures sex workers might face in their domestic lives. Despite selling sex for a living, sex workers have the same needs for nurturing, motherhood, romantic partners and a ‘normal’ domestic life as other women [19]. Condom use with main partners is notably lower than condom use with paying clients [1] and is an important counselling topic. In cases where sex workers disclosed their sex work occupation to their partners, index HIV testing will increase case finding of main partners and children for linkage to care [28].

Levels of contraception use are a major concern. With high rates of condom failure, clients sometimes insisting on condomless sex, and low condom use with main partners, sex workers need access to more effective contraception methods, such as hormonal implants. Limited access to modern contraceptives leads to unwanted and unintended pregnancies, as supported by the high proportion of planned abortions in our study. Comprehensive family planning services, including regular pregnancy testing and information on termination of pregnancy services, are clearly needed in our population. It is possible that having a child motivates sex workers to adopt safer behaviours that promote healthy lifestyles, stability and education for the children. For example, marijuana use was lower in women with one or more children. We propose standard enquiry about children and their wellbeing, and offering referrals for child support grants to assist sex workers that are mothers [29].

South Africa’s national network of dedicated sex worker services has already been shown to be acceptable to women [30]. Trained staff who are sensitised to the medical, emotional and legal needs of sex workers have been able to create user-friendly environments that facilitate women’s use of services. This could extend to the provision of cross-border services which promote retention in HIV treatment programmes [17].

Limitations of the study include the reliance on self-report, the cross-sectional nature of the study and the smaller sample size in Pretoria. The smaller sample in Pretoria has a two-fold explanation: firstly the programme only started in 2014 and it may take time for sex workers to become aware of the programme and enter its services. Also, the estimated sex worker population in Pretoria (13,218) is smaller than that in Johannesburg (21,540) [31]. Only sex workers that had been reached with services were included; those not accessing services are likely to differ from the study population in important ways. Missing these women and sub-populations of sex workers such as those who are internet-based likely diminishes the generalizability of the results. Also, other contributors to poor sexual health outcomes were not assessed, such as mental health status and experience of violence. Further, data were missing for some variables; likely as data were collected primarily for patient care and not for research purposes. However, the central role played in the programme by experienced peer educators and the use of sensitized clinical staff, may have strengthened the validity of the data.

Future research should explore individual risk profiling based on the typology of sex workers, and the specific vulnerabilities of sub-groups. As street-based sex workers are the most vulnerable sub-group, we propose research on harm reduction strategies to protect them from police arrest, public harassment and abuse [17]. Research on risk mitigation strategies used by sex workers could add new interventions to current programmes. Further, a better understanding of treatment cascades for sex workers and ways to reduce fall-off in the continuum of care [32] will support South Africa to achieve its ambitious HIV prevention targets [33].

## Conclusions

This study clearly shows the diversity of sex workers and their varying needs in the workplace and at home. Early engagement and consultation with sex workers in local sites is required to account for heterogeneity. The use of standardised treatment guidelines and a non-differentiated approach to care could seriously curtail the impact of the South African national sex work HIV Plan.

## Abbreviations

PrEP: Pre-Exposure Prophylaxis
SA: South Africa
SRH: Sexual and Reproductive Health
STI: Sexually Transmitted Infections

## References

[1] UCSF, Anova Health Institute, Wits, RHI: South African health monitoring survey (SAHMS), final report. The integrated biological and behavioural survey among female sex workers, South Africa 2013–2014. 2015. http://sanac.org.za/2016/03/29/ucsf-south-african-health-monitoring-survey-2016/. Accessed 22 Mar 2017.

[2] Dhana A, Luchters S, Moore L, Lafort Y, Roy A, Scorgie F, Chersich M (2014). Systematic review of facility-based sexual and reproductive health services for female sex workers in Africa. Glob Health.

[3] Scambler G, Paoli F (2008). Health work, female sex workers and HIV/AIDS: global and local dimensions of stigma and deviance as barriers to effective interventions. Soc Sci Med.

[4] Baral S, Beyrer C, Muessig K, Poteat T, Wirtz AL, Decker MR, Sherman SG, Kerrigan D (2012). Burden of HIV among female sex workers in low-income and middle-income countries: a systematic review and meta-analysis. Lancet Infect Dis.

[5] Soohoo M, Blas M, Byraiah G, Carcamo C, Brown B (2013). Cervical HPV infection in female sex workers: a global perspective. Open AIDS J.

[6] Bekker L-G, Johnson L, Cowan FM, Overs C (2015). Combination HIV prevention for female sex workers: what is the evidence?. Lancet.

[7] Luchters S, Bosire W, Feng A, Richter ML, King’ola N, Ampt F, Temmerman M, Chersich MF (2016). A baby was an added burden: predictors and consequences of unintended pregnancies for female sex workers in Mombasa, Kenya: a mixed-methods study. PLoS One.

[8] Saggurti N, Jain AK, Sebastian MP, Singh R, Modugu HR, Halli SS, Verma RK (2012). Indicators of mobility, socio-economic vulnerabilities and HIV risk behaviours among mobile female sex workers in India. AIDS Behav.

[9] Lafort Y, Lessitala F, Candrinho B, Greener L, Greener R, Beksinska M, Smit JA, Chersich M, Delva W (2016). Barriers to HIV and sexual and reproductive health care for female sex workers in Tete, Mozambique: results from a cross-sectional survey and focus group discussions. BMC Public Health.

[10] Vassall A, Chandrashekar S, Pickles M, Beattie TS, Shetty G, Bhattacharjee P, Boily M-C, Vickerman P, Bradley J, Alary M (2014). Community mobilisation and empowerment interventions as part of HIV prevention for female sex workers in southern India: a cost-effectiveness analysis. PLoS One.

[11] World Health Organization. Preventing HIV among sex workers in sub-Saharan Africa: a literature review; 2011. p. 35-35. http://www.who.int/iris/handle/10665/44549. Accessed 25 Sept 2016.

[12] Richter M, Chersich MF, Temmerman M, Luchters S (2013). Characteristics, sexual behaviour and risk factors of female, male and transgender sex workers in South Africa. S Afr Med J.

[13] UNAIDS. 90–90-90 an ambitious treatment target to help end the AIDS epidemic; 2014. p. 40-40. http://www.unaids.org/sites/default/files/media_asset/90-90-90_en.pdf. Accessed 22 July 2016.

[14] South African National AIDS Council. The south African national sex worker HIV plan, 2016–2019; 2016. p. 1–56. http://sanac.org.za/wp-content/uploads/2016/03/South-African-National-Sex-Worker-HIV-Plan-2016-2019-FINAL-Launch-Copy....pdf. Accessed 23 July 2016.

[15] World Health Organization. Guidelines on when to start antiretroviral therapy and on pre-exposure prophylaxis for HIV; 2015. p. 1–78. http://www.who.int/hiv/pub/guidelines/earlyrelease-arv/en/. Accessed 17 July 2016. 26598776

[16] Richter M, Chersich MF, Vearey J, Sartorius B, Temmerman M, Luchters S (2014). Migration status, work conditions and health utilization of female sex workers in three south African cities. J Immigr Minor Health.

[17] Scheibe A, Richter M, Vearey J, Padarath A, King J, Mackie E-L, Casciola J (2016). Sex work and South Africa’s health system: addressing the needs of the underserved. South African health review.

[18] Scorgie F, Nakato D, Akoth DO, Netshivhambe M, Chakuvinga P, Nkomo P, Abdalla P, Sibanda S, Richter M (2011). I expect to be abused and I have fear: sex workers’ experiences of human rights violations and barriers to accessing healthcare in four African countries.

[19] Basu A, Dutta MJ (2011). We are mothers first: Localocentric articulation of sex worker identity as a key in HIV/AIDS communication. Women Health.

[20] Scheibe A, Drame FM, Shannon K (2012). HIV prevention among female sex workers in Africa. Sahara J.

[21] Steen R, Chersich MF, de Vlas SJ (2012). Periodic presumptive treatment of curable sexually transmitted infections among sex workers: recent experience with implementation. Curr Opin Infect Dis.

[22] Harrison A, Newell M-L, Imrie J, Hoddinott G (2010). HIV prevention for south African youth: which interventions work? A systematic review of current evidence. BMC Public Health.

[23] Stadler J, Delany S (2006). The 'healthy brothel': the context of clinical services for sex workers in Hillbrow South Africa. Cult Health Sex.

[24] Wojcicki JM (2002). Commercial sex work or Ukuphanda? Sex-for-money exchange in Soweto and Hammanskraal area, South Africa. Cult Med Psychiatry.

[25] Hao C, Liu H, Sherman SG, Jiang B, Li X, Xu Y, Jiang Z, Zang C (2014). Typology of older female sex workers and sexual risk for HIV infection in China: a qualitative study. Cult Health Sex.

[26] Goldenberg SM, Chettiar J, Nguyen P, Dobrer S, Montaner J, Shannon K (2014). Complexities of short-term mobility for sex work and migration among sex workers: violence and sexual risks, barriers to care, and enhanced social and economic opportunities. J Urban Health.

[27] Wechsberg WM, Luseno WK, Lam WKK, CDH P, Morojele NK (2006). Substance use, sexual risk, and violence: HIV prevention intervention with sex workers in Pretoria. AIDS Behav.

[28] National Department of Health (2015). National HIV Counselling and testing (HCT) policy guidelines.

[29] Sloss CM, Harper GW (2004). When street sex workers are mothers. Arch Sex Behav.

[30] Lince-Deroche N, Pleaner M, Harries J, Morroni C, Mullick S, Firnhaber C, Mulongo M, Holele P, Sinanovic E: Achieving universal access to sexual and reproductive health services: the potential and pitfalls for contraceptive services in South Africa. In SA health review. Edited by Padarath A, King J, Mackie E-L, Casciola J. Pretoria: Health Systems Trust; 2016:95-108.

[31] South African National AIDS Council: Estimating the size of the sex worker population in South Africa. 2013. http://www.sweat.org.za/wp-content/uploads/2016/02/Sex-Workers-Size-Estimation-Study-2013.pdf. Accessed 18 July 2016.

[32] Ayala G, Makofane K, Santos GM, Arreola S, Hebert P, Thomann M, Wilson P, Beck J, Do TD (2014). HIV treatment cascades that leak: correlates of drop-off from the HIV care continuum among men who have sex with men worldwide. J AIDS Clin Res.

[33] South African National AIDS Council. South African national strategic plan on HIV, STIs and TB 2012–2016; 2011. p. 84-84. http://www.hst.org.za/publications/national-strategic-plan-hiv-stis-and-tb-2012-2016. Accessed 23 Aug 2016.

